# Exploring New Inflammatory Biomarkers and Pathways during LPS-Induced M1 Polarization

**DOI:** 10.1155/2016/6986175

**Published:** 2016-12-21

**Authors:** Carolina Cunha, Cátia Gomes, Ana Rita Vaz, Dora Brites

**Affiliations:** ^1^Research Institute for Medicines (iMed.ULisboa), Faculty of Pharmacy, Universidade de Lisboa, Lisbon, Portugal; ^2^Department of Biochemistry and Human Biology, Faculty of Pharmacy, Universidade de Lisboa, Lisbon, Portugal

## Abstract

Identification of mediators triggering microglia activation and transference of noncoding microRNA (miRNA) into exosomes are critical to dissect the mechanisms underlying neurodegeneration. We used lipopolysaccharide- (LPS-) induced N9 microglia activation to explore new biomarkers/signaling pathways and to identify inflammatory miRNA (inflamma-miR) in cells and their derived exosomes. Upregulation of iNOS and MHC-II (M1-markers) and downregulation of arginase 1, FIZZ1 (M2-markers), and CX3CR1 (M0/M2 polarization) confirmed the switch of N9 LPS-treated cells into the M1 phenotype, as described for macrophages/microglia. Cells showed increased proliferation, activated TLR4/TLR2/NF-*κ*B pathway, and enhanced phagocytosis, further corroborated by upregulated MFG-E8. We found NLRP3-inflammasome activation in these cells, probably accounting for the increased extracellular content of the cytokine HMGB1 and of the MMP-9 we have observed. We demonstrate for the first time that the inflamma-miR profiling (upregulated miR-155 and miR-146a plus downregulated miR-124) in M1 polarized N9 cells, noticed by others in activated macrophages/microglia, was replicated in their derived exosomes, likely regulating the inflammatory response of recipient cells and dissemination processes. Data show that LPS-treated N9 cells behave like M1 polarized microglia/macrophages, while providing new targets for drug discovery. In particular, the study yields novel insights into the exosomal circulating miRNA during neuroinflammation important for emerging therapeutic approaches targeting microglia activation.

## 1. Introduction

Microglia are a unique cell population within the central nervous system (CNS) as they descend from myeloid origin and are commonly recognized as the resident immune cells in the brain [1]. They constitute about 10–20% of glial cell population and are continuously monitoring the surrounding environment, acting as sensors of CNS homeostasis [2, 3]. Microglia rapidly change their morphology, gene expression, and functional performance upon any threat to tissue homeostasis, acquiring an activated phenotype, which is an adaptive process specific for each stimulus and CNS region [4]. Accumulating evidence supports their involvement in synaptic development and remodeling [5], emphasizing that microglial functions are extended beyond immune-defense mechanisms. Although microglial cells are first protectors of brain homeostasis, in case of prolonged or chronic stimulation they may become deleterious to the neuronal population. Indeed, exacerbated stages of neurotoxicity can progress to pathological conditions including neurodegenerative disorders such as Alzheimer's disease or Parkinson's disease [6], where microglia actively contributes to neuroinflammation and neuronal degeneration [7].

Despite the diversity of microglia responses, their activation has been characterized by a recognized number of phenotypes classically described for macrophages [8]. The surveillant/nonpolarized phenotype, also known as M0, describes alert but not activated microglia which are continuously screening the environment [9]. The almost exclusive microglial fractalkine receptor, CX3C chemokine receptor 1 (CX3CR1), is highly expressed in M0 phenotype [10] but, besides the maintenance of microglia surveillance, CX3CR1/fractalkine cross talk is also important in promoting migration of activated cells [11]. The M1 phenotype or classical activated microglia can be induced by lipopolysaccharide (LPS) or interferon-gamma (IFN-*γ*) with increased production of proinflammatory cytokines, chemokines, matrix metalloproteinases (MMPs), as well as reactive oxygen and nitrosative species (ROS and RNS, resp.), among others [12]. M1 microglia is associated with a neurotoxic phenotype with enhanced major histocompatibility complex class II (MHC-II), inducible nitric oxide synthase (iNOS/NOS2), and interleukin-1*β* (IL-1*β*) markers. The alternative M2 phenotype that is related to the damage resolution [13] may include several subtypes [7] and is induced by IL-4, IL-10, and transforming growth factor-*β* (TGF-*β*). Arginase 1 (arg1), found in inflammatory zone 1 (FIZZ1), and Ym1 are recognized markers of M2 polarization [14]. However, although the expression of these markers is used to differentiate microglia phenotypes, there is still much to learn about the determinants of microglia specific functional polarization.

Stimulation of the toll-like receptors (TLRs) signaling cascade is known to trigger the translocation of nuclear factor kappa B (NF-*κ*B) into the nucleus and the expression of proinflammatory genes [15], involving the activation of the inflammasome. However, its activation in the context of microglia neurotoxic potential remains unknown. Inflammasome mediators comprise NOD-like receptor family, pyrin domain containing 3 (NLRP3) and caspase-1 responsible for the cleavage of IL-18 and IL-1*β* proforms [16]. Recently, it was shown that the release of the alarmin high mobility group box 1 (HMGB1) is mediated by the NLRP3 inflammasome activation [17] and constitutes a signal to activate microglia [18], though the regulation process is still unclear.

Together with the release of inflammatory mediators, microglia migration and phagocytosis are part of the cell response to injury. Protein milk fat globule-EGF factor 8 (MFG-E8) was shown to recognize phosphatidylserine (PS) in the apoptotic neurons, thus enabling microglial phagocytosis [19]. Nevertheless, its specific regulation in different challenging situations remains unknown.

The majority of these inflammatory pathways have been identified along diverse studies performed with macrophage/microglia primary cultures. Due to such culture time consumption and reduced yield for the experimental assays, all the collected information on microglia inflammatory mediators is fragmented. Therefore, we here embraced the assessment of an integrated study on the several inflammatory signaling pathways leading to the upregulation of microglia M1 polarization biomarkers and downregulation of those related to M2 subtypes in the microglial N9 cells upon LPS treatment. N9 cells were generated by immortalization of embryonic primary cultures from the ventral mesencephalon and cerebral cortex of ICR/CD1 mice using oncogenic murine retroviruses carrying the v-myc or the v-mil oncogenes of the avian retrovirus MH2 [20]. These cells have been preferentially used due to the simplicity and ease of manipulation, but only a limited number of inflammatory mediators and genes were identified in N9 cells, despite responding similarly to LPS as primary microglial cells derived from the same mouse strain [21].

MicroRNAs (miRNAs) have recently emerged as key regulators of inflammation and as mediators of macrophage/microglia polarization [22]. Actually the inflamma-miRs, miR-155, and miR-146a have been related to the microglia polarization into M1. While the first enhances the proinflammatory response, the second acts as a negative regulator [23] being essential in halting excessive inflammation. Oppositely, miR-124, miR-21, and miR-145 are associated with an anti-inflammatory response repressing the M1 phenotype polarization [24]. However, it is accepted that such microglia phenotype regulation is quite complex and miR-146a, as an example, may be increased during M1 microglia polarization being also overexpressed in dystrophic/senescent macrophages [25], whereas miR-124 has been identified in surveillant microglia, as well as in M2 microglia [26]. Another issue that has been recently addressed is the particular importance of the exosomes for sustained inflammation. Exosomes are small vesicles (~100 nm) formed through the endocytic process and released upon multivesicle bodies fusion with the plasma membrane [27, 28]. They have been associated with intercellular communication, even at long distances, by direct transfer of mRNA, proteins, and miRNAs, the last being essential for regulating gene expression in the recipient cells.

Since the pathways underlying the switch of microglia towards the M1 phenotype are not fully understood, we first characterized the polarization of N9 microglial cells into the M1 subtype upon LPS exposure, based on macrophage/microglia M1 and M2 biomarkers, and consequent microglia innate functions, such as phagocytosis and chemotaxis. Much attention has been lately given on microglia-dependent inflammasome activation [29, 30], but no data are available on LPS-treated microglia, which is the reason why we assessed the inflammasome multiprotein complex in our model. Once miRNAs are emerging as potent fine-tuners of neuroinflammation [31] and indicated to regulate the inflammatory response when transported in exosomes from primary bone marrow-derived dendritic cells [32], we decided to assess their representation in the LPS-polarized cells and in their derived exosomes to extend our knowledge on such issue, still scarcely explored in microglia primary cultures and unknown in N9 cells. Actually, exosomal miRNAs are currently being extensively studied as biomarkers of disease and the understanding on how they are loaded into exosomes and delivered to specific recipient cells may help in developing therapeutic approaches to modulate innate cell function. Here, we have further clarified microglia inflammatory mediators and targets that once modulated may restrict microglia activation in neurodegenerative disorders, like Alzheimer's disease and amyotrophic lateral sclerosis.

## 2. Materials and Methods

### 2.1. N9 Cell Culture and Treatment

N9 cell line was a gift from Teresa Pais (Institute of Molecular Medicine, Universidade de Lisboa, Portugal). Cells (8.3 × 10^4^ cells/cm^2^) were plated on uncoated 12- or 6-well tissue culture plates (Orange Scientific, Braine-l'Alleud, Belgium) in culture medium [RPMI media supplemented with fetal bovine serum (FBS) (10%) and L-glutamine (1%) and with the antibiotic penicillin/streptomycin (1%)] and were grown to confluence before experiments. No bacterial contaminations were observed in any experiment. To induce N9 cells reactivity we used 300 ng/mL of lipopolysaccharide (LPS,* E. coli* O111:B4, 437627, Calbiochem, Darmstadt, Germany) diluted in basal media for 24 h, as described by Cui and colleagues [33]. Response of LPS-treated cells was compared with nontreated microglia (control).

### 2.2. Determination of Cell Death

Phycoerythrin-conjugated annexin V (V-PE) and 7-aminoactinomycin-D (7-AAD) mixture (Guava Nexin® Reagent, #4500-0450, Millipore, Billerica, MA, USA) were used to determine the percentage of viable, early-apoptotic, and late-apoptotic/necrotic cells by flow cytometry. After incubation, adherent microglia were collected by trypsinization and added to the cells present in the incubation media. After centrifugation, the pellet of cells was resuspended in PBS containing 1% of bovine serum albumin (BSA), stained with Guava Nexin Reagent according to manufacturer's instruction, and analyzed on a Guava easyCyte 5HT flow cytometer (Guava Nexin Software module, Millipore), as usual in our lab [34]. Two readings were performed for each sample.

### 2.3. Quantitative RT-PCR

After incubation, cellular media were removed and cells were collected with TRIzol® (Life Tecnologies, Carlsbad, CA, USA) using a cell scrapper as implemented in the lab [35]. Total RNA was then extracted from N9 cells using TRIzol reagent method according to manufacturer's instructions and quantified using Nanodrop ND-100 Spectrophotometer (NanoDrop Technologies, Wilmington, DE, USA). Conversion into cDNA was performed with RevertAid H Minus First Strand cDNA Synthesis Kit (Thermo Scientific, Waltham, MA, USA). Quantitative RT-PCR (qRT-PCR) was performed by using *β*-actin as an endogenous control to normalize the expression level. The sequences used for primers are represented in Table S1 (Supplementary Data, in Supplementary Material available online at http://dx.doi.org/10.1155/2016/6986175). qRT-PCR was accomplished on a 7300 Real-Time PCR System (Applied Biosystems, Life Technologies) using a SYBR Green qPCR Master Mix (Thermo Scientific). The qRT-PCR was performed in 96-well plates with each sample performed in triplicate, and no-template control was included for each amplification. qRT-PCR was achieved under optimized conditions: 52°C for 2 min followed by 95°C for 10 min and finally 40 cycles at 95°C for 0.15 min and 62°C for 1 min. In order to verify the specificity of the amplification, a melt-curve analysis was performed, immediately after the amplification protocol. Nonspecific products of PCR were not found in any case. Relative mRNA concentrations were calculated using the ΔΔCT equation. For miRNA analysis, conversion of cDNA was achieved with the universal cDNA Synthesis Kit (Exiqon, Vedbaek, Denmark) as described by Cardoso et al. [36], following manufacturer's recommendations. The miRCURY LNA™ Universal RT miRNA PCR system (Exiqon) was used in combination with predesigned primers (Exiqon), represented in Table S1 (Supplementary Data), using SNORD110 as reference gene. The reaction conditions consisted of polymerase activation/denaturation and well-factor determination at 95°C for 10 min, followed by 50 amplification cycles at 95°C for 10 s and 60°C for 1 min (ramp-rate 1.6°/s). Relative mRNA concentrations were calculated using the ΔΔCT equation. Two readings were performed for each sample.

### 2.4. Western Blot Analysis

After incubation, cellular media were removed and cells collected with Cell Lysis Buffer (Cell Signaling, Beverly, MA, USA) plus 1 mM phenylmethylsulfonyl fluoride (PMSF, Sigma) as usual in our lab [37]. Briefly, total cell extracts were lysed for 5 minutes on ice with shaking, collected with cell scrapper, and sonicated for 20 secs. The lysate was then centrifuged at 14,000*g* for 10 min at 4°C, and the supernatants were collected and stored at −80°C. Nuclear extracts were prepared as usual in our lab [37]. Protein content in the extracellular media was obtained by precipitation using trichloroacetic acid in 10% (v/v) of acetone solution. After a centrifugation of 15,000*g* for 10 min at 4°C, the pellet was washed in acetone containing 10 mM dithiothreitol resuspended in lysis buffer and stored at −80°C. Protein concentration in total and nuclear extracts, as well as in extracellular medium, was determined using a protein assay kit (Bio-Rad, Hercules, CA, USA) according to manufacturer's specifications. Then, equal amounts of protein were subject to SDS-PAGE and transferred to a nitrocellulose membrane. After blocking with 5% (w/v) nonfat milk solution, membranes were incubated with primary antibodies (Supplementary Data, Table S2) diluted in 5% (w/v) BSA overnight at 4°C, followed by the secondary antibodies goat anti-rabbit HRP-linked (1 : 5000, sc-2004, Santa Cruz Biotechnology®, CA, USA) and goat anti-mouse HRP-linked (1 : 5000, sc-2005, Santa Cruz Biotechnology) diluted in blocking solution. Chemiluminescence detection was performed by using LumiGLO® reagent (Cell Signaling) and bands were visualized in the ChemiDoc™ XRS System (Bio-Rad). The relative intensities of protein bands were analyzed using the Image Lab™ analysis software (Bio-Rad). One single reading was performed for each sample.

### 2.5. Immunocytochemistry

For immunofluorescence detection, N9 cells were fixed with freshly prepared 4% (w/v) paraformaldehyde in PBS and a standard immunocytochemical technique was performed as previously indicated [37]. Briefly, cells were incubated overnight at 4°C with the primary antibodies: rabbit anti-Iba1 (1 : 250, 019-19741, Wako, Wako Pure Chemical Industries Ltd., Osaka, Japan), rabbit anti-NF-*κ*B (1 : 500 or 1 : 200 for nuclear extracts, sc-372, Santa Cruz Biotechnology), and goat anti-Ki-67 (1 : 50, Santa Cruz Biotechnology, sc-7846). The secondary antibodies incubated for 2 h at room temperature were goat anti-rabbit Alexa Fluor 488, goat anti-rabbit Alexa Fluor 594, and rabbit anti-goat 594 (1 : 1000, Invitrogen Corporation™, Carlsbad, CA, USA). Cell nuclei were stained with Hoechst 33258 dye (blue, Sigma-Aldrich). Fluorescence was visualized using an AxioCam HR camera adapted to an AxioScope A1® microscope (Zeiss, Germany). Merged images of UV and fluorescence of ten random microscopic fields were acquired per sample by using Zen 2012 (blue edition, Zeiss) software. Original magnifications used were 400 and 630x.

For morphological characterization of N9 microglia, we used the particle measurement analysis in ImageJ (1.47v, NIH, USA) to automatically measure the 2D area, perimeter, and Feret's diameter of single microglia cells after Iba-1 immunostaining, which are considered valuable additional parameters to evaluate the shape of microglia [38]. As indicated in Figure S1, we observed that ramified N9 microglia presented lower number of ramifications in comparison to primary cultured microglia (Fig. S1B) obtained from mice cortex [39]. Nevertheless, we were able to observe different N9 microglia morphologies that included round/oval shape (Fig. S1B), ramified with 2 or 3 processes (Fig. S1C-D), and amoeboid shape, either completely devoid of ramifications or with thicker and shorter branches (Fig. S1D-E). Translocation of NF-*κ*B from the cytoplasm to the nucleus was determined by the quantification of the number of NF-*κ*B-positive nuclei and normalized to the total number of cells. For evaluation of the proliferation ability of microglia, we quantified the number of cells showing Ki-67 expression in the nucleus. Ki-67 is present in the nucleus during all phases of the cell cycle while being absent in the resting stage (G0).

### 2.6. Quantification of Nitrite Levels

NO levels were estimated by assessing the concentration of nitrites (NO_2_), the stable end-product from NO metabolism, in culture media by the Griess method, as we published [40]. Briefly, extracellular media free from cellular debris were mixed with Griess reagent in 96-well tissue culture plates for 10 min in the dark, at RT. The absorbance at 540 nm was determined using a microplate reader. A calibration curve was used for each assay. All samples were measured in duplicate and the mean value was used. Two readings were performed for each sample.

### 2.7. Gelatin Zymography

MMP-9 and MMP-2 activities were determined in the N9 extracellular media by performing a SDS-PAGE zymography in 0.1% gelatin-10% acrylamide gels, under nonreducing conditions, as previously described [41]. After electrophoresis, the gels were washed for 1 h with 2.5% Triton-X-100 in 50 mM Tris pH 7.4 containing 5 mM CaCl_2_ and 1 *μ*M ZnCl_2_, to remove SDS and to renature the MMP species in the gel. To induce gelatin lysis, the gels were incubated at 37°C in the developing buffer (50 mM Tris pH 7.4, 5 mM CaCl_2_, 1 *μ*M ZnCl_2_) overnight. For enzyme activity analysis, the gels were stained with 0.5% Coomassie Brilliant Blue R-250 (Sigma-Aldrich) and destained in 30% ethanol/10% acetic acid/H_2_O (v/v). Gelatinase activity, detected as a white band on a blue background, was measured using computerized image analysis (Image Lab, Bio-Rad). One reading was performed for each sample.

### 2.8. Caspase-1 Activity Assay

Activity of caspase-1 was determined by a colorimetric method (Calbiochem, Darmstadt, Germany) as published by us [42]. Briefly, cells were harvested, washed with ice-cold PBS, and lysed for 30 min on ice in the lysis buffer. The activity of caspase-1 was assessed in cell lysates by enzymatic cleavage of chromophore pNA from the substrate, according to manufacturer's instructions. The proteolytic reaction was carried out in protease assay buffer containing 2 mM Ac-YVAD-pNA. Following incubation of the reaction mixtures, the formation of pNA was measured at *λ* = 405 nm with a reference filter of 620 nm. One reading was performed for each sample.

### 2.9. Microglial Phagocytosis Assay

To evaluate the phagocytic ability of N9 microglia, cells were incubated with 0.0025% (w/w) fluorescent latex beads, diameter 1 *μ*m, for 75 min at 37°C and fixed with freshly prepared 4% (w/v) paraformaldehyde in PBS. N9 cells were immunostained with rabbit anti-Iba1 (1 : 250, 019-19741, Wako), and nuclei were counterstained with Hoechst 33258 dye (blue). UV and fluorescence images of ten random microscopic fields (original magnification: 400x) were acquired per sample by using Zen 2012 (blue edition, Zeiss) software. Total phagocytic cells and the number of ingested beads per cell were counted with ImageJ software to determine the percentage of phagocytic cells and the mean number of ingested beads per cell [41].

### 2.10. Cell Migration Assay

Cell migration assay was performed in a 48-well microchemotaxis chamber (Boyden Chamber, Neuro Probe, Gaithersburg, MD, USA) as we published [39]. Briefly, N9 cells were resuspended in serum-free RPMI and 50 *μ*L of cell suspension was placed into each top well (2–4 × 10^4^ cells per well). The bottom wells were filled with serum-free RPMI (basal medium) alone, or with ATP (10 *µ*M), and LPS (300 ng/mL) diluted in basal medium. Microglial cells were allowed to migrate for 6 h through a polycarbonate track-etch membrane with polyvinylpyrrolidone (PVP) (Neuro Probe) towards the solution in the bottom wells. Afterwards, the membrane was removed and the bottom side fixed with cold methanol. Cells were stained with 10% Giemsa in PBS (w/v), freshly prepared and filtered. The number of total cells was counted in ten microscopic fields with ImageJ software (original magnification: 100x) acquired to observe the complete well using Leica IM50 software and Leica DFC490 camera (Leica Microsystems, Wetzlar, Germany), adapted to an AxioSkope HBO50 microscope (Zeiss). For each experiment, at least three wells per condition were acquired.

### 2.11. Exosome Isolation

Exosomes were obtained from the extracellular media of N9 cells either from control or from LPS-stimulated cells, according to Wang et al. [43], with minor modifications. Briefly, 20 mL of extracellular media was centrifuged at 1,000*g* for 10 min to remove dead cells and debris followed by another centrifugation at 16,000*g* for 60 min, to separate microvesicles (size ~1000 nm). The recovered supernatant was passed through a 0.2 *µ*m filter to remove suspended particles and further centrifuged in the Ultra L-XP100 centrifuge (Beckman Coulter Inc., California, USA) at 100,000*g* for 120 min. This pellet fraction (exosomes, size ~100 nm) was resuspended in PBS and centrifuged again at 100,000*g* for 120 min. The final pellet containing exosomes was resuspended in lysis buffer and RNA inside exosomes was extracted using miRCURY Isolation Kit-Cell (Exiqon), according to manufacturer's instructions. Briefly, after lysis of the exosomes, the RNA was absorbed to a silica matrix, washed with the recommended buffers, and eluted with 20 *µ*L of the supplied elution buffer by centrifugation. The synthesis of cDNA and RT-PCR was performed as mentioned above.

### 2.12. Dynamic Light-Scattering (DLS) Measurements

Size measurements were made at 25°C with a Zetasizer Nano S DLS apparatus (Malvern Instruments, Worcestershire, UK). After the ultracentrifugation procedure abovementioned, each sample was diluted in PBS and was read three times in the Zeta Sizer Nano S (Zen 1600) to evaluate the diameter of the collected particles. A histogram of the percentage of particles with specific diameters was calculated using DTS (nano) 7.03 software (Malvern Instruments). Data represent an average of 3 measurements for each sample.

### 2.13. Statistical Analysis

The results of at least four independent experiments are expressed as mean ± s.e.m. Comparisons between LPS-treated N9 cells and nontreated cells (control) in all experiments were made via two-tailed Student's *t*-test or unpaired *t*-test with Welch's correction, depending on whether variances were equal or different, respectively. Comparison of more than two groups, namely, in the morphological characterization, chemotaxis assay, and phagocytosis tests, was done by one-way ANOVA followed by multiple comparisons Bonferroni post hoc correction using GraphPad Prism 5 (GraphPad Software, San Diego, CA, USA). Values of *p* < 0.05 were considered statistically significant and those of *p* < 0.01 and *p* < 0.001 highly significant.

## 3. Results

### 3.1. LPS-Treated N9 Microglia Acquire a M1 Phenotype, with Upregulation of Specific M1-Markers, with Main Amoeboid Morphology and Increased Proliferation Rate

**Figure 1:**
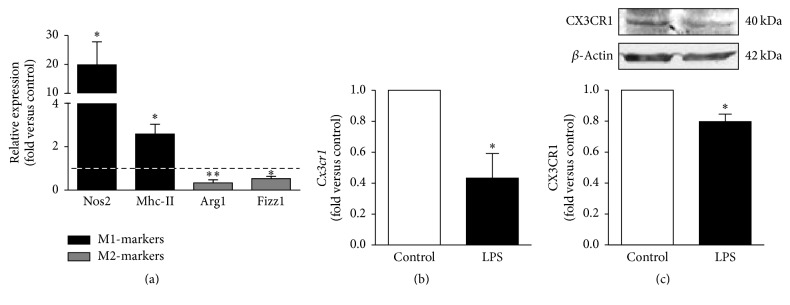
Lipopolysaccharide (LPS) polarizes N9 microglia into M1 rather than M2 phenotype. Expression of genes [inducible nitric oxide synthase (*Nos2*), major histocompatibility complex class II (*Mhc-II*), arginase 1 (*Arg1*), found in inflammatory zone 1 (*Fizz1*) (a), and CX3C chemokine receptor 1 (*Cx3cr1*)] (b) was determined by qRT-PCR (each sample was evaluated in duplicate). Markers of classically (M1) and alternatively activated (M2) microglia are indicated in black and gray, respectively. CX3CR1 protein levels were assessed by Western blot analysis (c). Comparisons between LPS-treated N9 cells and nontreated cells (control) were made via two-tailed Student's *t*-test. Results are mean ± s.e.m. from five (a, c) or six (b) independent experiments. Results from Western blot (c) were performed in duplicate. ^*∗*^ *p* < 0.05 and ^*∗∗*^ *p* < 0.01 versus nontreated cells (control, assumed value of 1, dashed line).

Despite the great plasticity of microglial responses, the different subsets of microglia activation have been characterized by the expression of specific markers, as previously referred. Moreover, in addition to the well-known proinflammatory response mediated by TLR/NF-kB pathway, microglia activation may also imply the alteration of the gene expression profile not only by inducing the transcription of specific genes but also by downregulating nonrequired functions. We have explored the mRNA expression of M1 and M2 markers, as well as the fractalkine receptor CX3CR1 in N9 microglia exposed to LPS, not fully documented in previous studies. As shown in Figure 1, incubation of N9 microglia with LPS led to increased expression of the M1-markers* Nos2* and* Mhc-II*, while promoting the downregulation of the M2-markers* arg1* and* Fizz1* (Figure 1(a)). Expression of both mRNA and protein levels of CX3CR1 were found diminished by LPS exposure (Figures 1(b) and 1(c), resp.), indicating a reduced prevalence of cells with surveillant/anti-inflammatory properties, thus favoring a major representation of M1 polarized microglial cells.

**Figure 2:**
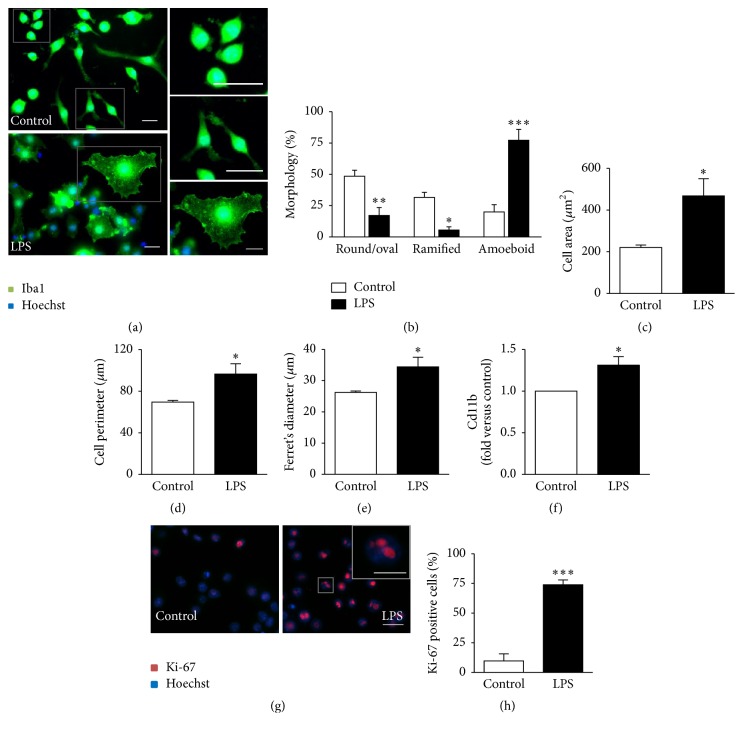
M1 polarized N9 microglia display amoeboid morphology, together with increased cell area, perimeter, and Feret's diameter, while showing increased proliferation rate based on the high representation of CD11b and Ki-67 positive cells. Morphological analysis and detection of Ki-67 in the nucleus were performed by immunocytochemistry using anti-Iba1 and anti-Ki-67, respectively, as indicated in Materials and Methods. For morphology, representative results of one experiment are shown (a) and quantified as the percentage of cells with different morphologies, namely, round/oval, ramified, and amoeboid (b). Evaluation of cell area (c), perimeter (d), and Feret's diameter (e) was performed using the computer program ImageJ.* Cd11b* expression was assessed by qRT-PCR (f). Representative results of Ki-67 immunostaining are shown (g) and expressed as the percentage of Ki-67 positive cells versus total number of cells (h). Results are mean ± s.e.m. from four independent experiments, except for* Cd11b* expression (f), where five experiments were performed. Comparison of more than two groups (b) was done by one-way ANOVA followed by multiple comparisons Bonferroni post hoc correction. Comparisons between lipopolysaccharide-treated (LPS-treated) N9 cells and nontreated cells (control) were made via two-tailed Student's *t*-test (f, h) or unpaired *t*-test with Welch's correction (c, d, e). ^*∗*^ *p* < 0.05, ^*∗∗*^ *p* < 0.01, and ^*∗∗∗*^ *p* < 0.001 versus nontreated cells (control). Scale bar represents 20 *µ*m (and 10 *µ*m in the close-up shown inset in (g)).

Morphological changes and increased cell density have been indicated for microgliosis [44]. Although insufficient to characterize microglial functional behavior, these parameters are still useful to illustrate microglia activation. Therefore, we have additionally performed a morphological characterization by immunocytochemistry using the microglia specific marker Iba1, together with cell area, perimeter, and Feret's diameter [38]. As depicted in Figure 2, our results showed that N9 microglia change from a round/oval or ramified morphology to an amoeboid shape, presenting either a complete absence of ramifications or the presence of thicker and shorter branches, in about 75% of the cells (*p* < 0.001) incubated with LPS (Figures 2(a) and 2(b)). We have previously shown that reactive isolated primary microglial cultures also show a similar morphology [39]. Accordingly, area, perimeter, and Feret's diameter were increased in LPS-treated cells (Figures 2(c), 2(d) and 2(e), resp.). In order to evaluate cell density, we evaluated the* Cd11b* expression and the number of cells presenting nuclear Ki-67 expression. We observed an increased* Cd11b* expression (Figure 2(f)) and a higher number of Ki-67-positive nuclei (Figures 2(g) and 2(h)) in LPS-stimulated cells as compared with control samples. Now, considering the cellular viability of N9 microglia after LPS exposure, we found that the number of viable cells was decreased (from 84% in control to 67% in LPS-treated N9 cells, *p* < 0.01, Table 1), as a consequence of the increased number of microglia showing early-apoptotic features.

### 3.2. M1 Polarized N9 Microglia Show Reduced Migration Ability Towards Chemoattractants but Display Increased Phagocytosis and MFG-E8 Expression

**Figure 3:**
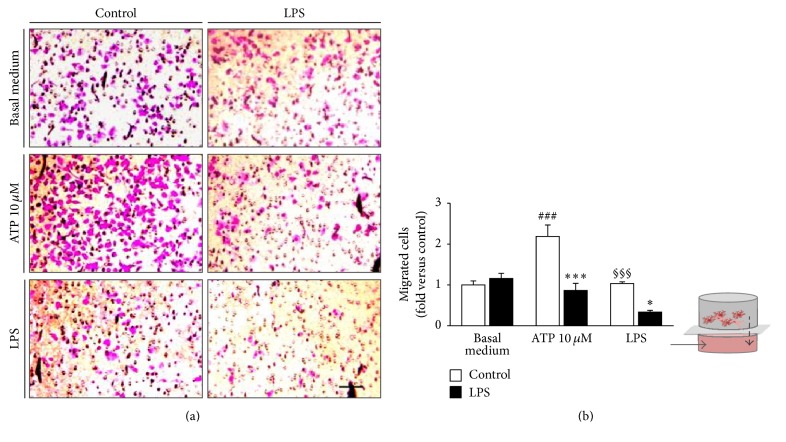
M1 polarized N9 microglia have reduced ability to migrate towards ATP and lipopolysaccharide (LPS). Migration was assessed using a Boyden Chamber. Nontreated (control) and LPS-stimulated microglia were allowed to migrate for 6 h to basal medium (basal migration), ATP at 10 *μ*M and LPS at 300 ng/mL. Representative results of one experiment are shown (a). The total number of cells per well was counted and the results were expressed as fold versus control (basal migration) (b). Results are mean ± s.e.m. from four independent experiments. Comparison was done by one-way ANOVA followed by multiple comparisons Bonferroni post hoc correction. ^*∗*^ *p* < 0.05 and ^*∗∗∗*^ *p* < 0.001 versus respective control; ^###^ *p* < 0.001 versus basal migration in controls; and ^§§§^ *p* < 0.001 versus migration of control cells to ATP. Scale bar represents 100 *μ*m.

After confirming the typical features of microglia M1 polarization in N9 cells, we wondered whether LPS was able to modify their migration ability. For that, we have performed a chemotaxis assay using the Boyden Chamber, and migration of LPS-treated and nontreated microglia was tested towards well-known chemoattractants, such as ATP (10 *µ*M) [3, 9] and LPS (300 ng/mL) [45]. Nontreated N9 cells were highly responsive to ATP chemoattraction (~2-fold increase, *p* < 0.001) in comparison to those freely migrating to the basal medium and to the LPS-treated cells that were revealed to become unresponsive (Figure 3). To note, however, is that LPS-treated cells showed the same migration ability as nontreated microglia towards the basal medium. LPS revealed a lower chemoattractive capacity when compared to that of ATP (*p* < 0.001) and, as observed for ATP, the LPS-treated cells also showed a reduced mobility as compared to cells that were not exposed to LPS stimulus.

**Figure 4:**
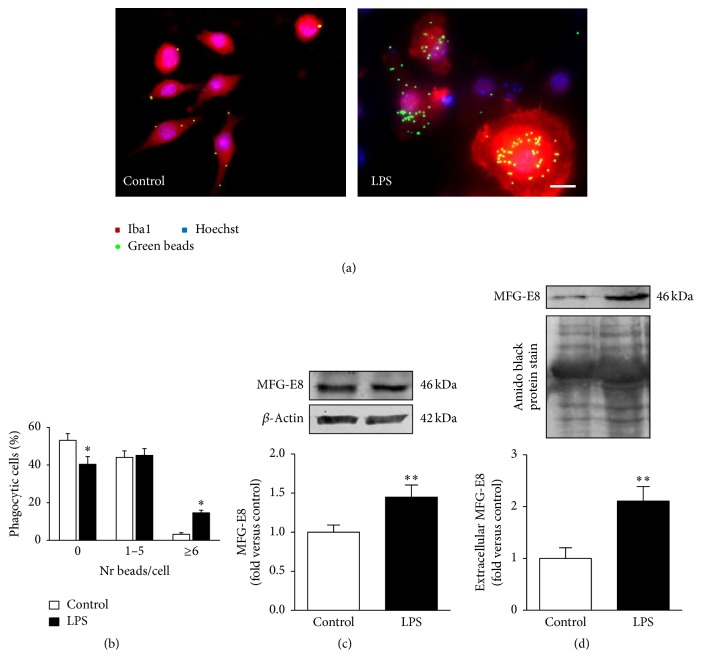
M1 polarized N9 microglia show enhanced phagocytic ability and milk fat globule-EGF factor 8 (MFG-E8) upregulated expression. Representative results of one experiment are shown for the phagocytosis of latex beads (a) and results are expressed as the percentage of cells engulfing specific numbers of ingested beads versus total number of cells (b). MFG-E8 expression and release were assessed by Western blot analysis ((c) and (d), resp.). Results are mean ± s.e.m. from five (b) or six (c, d) independent experiments, performed in duplicate. Comparison of more than two groups (b) was done by one-way ANOVA followed by multiple comparisons Bonferroni post hoc correction. Comparisons between lipopolysaccharide- (LPS-) treated N9 cells and nontreated cells (control) were made via two-tailed Student's *t*-test (c, d). ^*∗*^ *p* < 0.05 and ^*∗∗*^ *p* < 0.01 versus nontreated cells (control). Scale bar represents 20 *μ*m.

When analyzing the phagocytic properties of microglia, essential for clearance of debris and elimination of pathogenic organisms [46], the treatment with LPS reduced the number of cells showing no intracellular beads (*p* < 0.05), while increasing those with more than 6 ingested beads (*p* < 0.05) (Figures 4(a) and 4(b)). In addition, we were able to observe, for the first time in this model, that 24 h incubation with LPS induces the expression and release of MFG-E8 from N9 cells (~1.5- and ~2-fold, respectively, *p* < 0.01), as indicated by Western blot analysis of total extracts and extracellular media (resp., Figures 4(c) and 4(d)). We may then assume that LPS-treated cells intensify their phagocytic ability as a defensive mechanism despite their increased immobility.

### 3.3. M1 Polarization of Microglia is Mediated by Activation of the Inflammatory TLR2/TLR4/NF-kB/Inflammasome Signaling Cascade and Release of HMGB1

**Figure 5:**
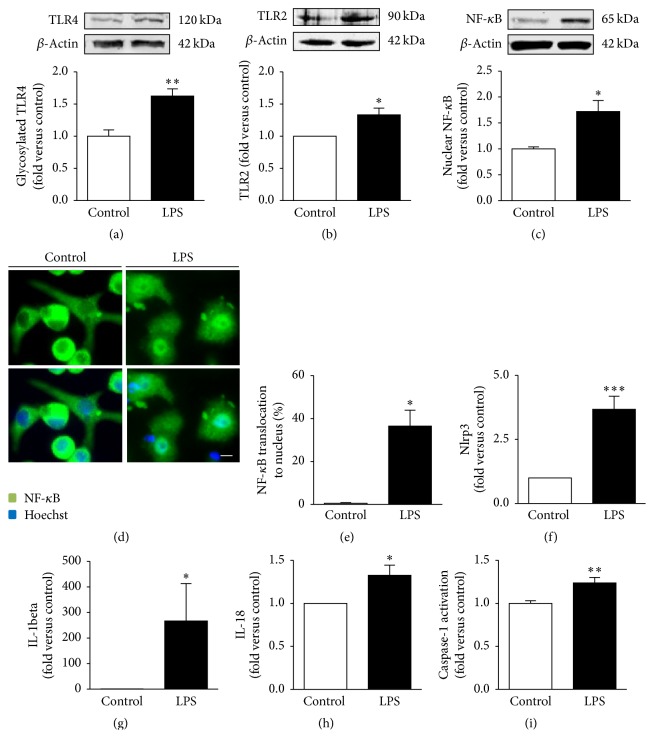
M1 polarized N9 microglia evidence activation of TLR4/TLR2/NF-*κ*B signaling pathway and of inflammasome complex. Protein expression of glycosylated toll-like receptor 4 (TLR4) (a) and TLR2 (b) was evaluated by Western blot analysis. Translocation of nuclear factor kappa B (NF-*κ*B) to the nucleus is represented by considering the Western blot analysis of nuclear expression (c). To observe NF-*κ*B cellular localization, immunocytochemistry against anti-NF-*κ*B was performed as indicated in Materials and Methods and representative results are shown (d) and presented as the percentage of cells with nuclear staining versus total number of cells (e). NOD-like receptor family, pyrin domain containing 3* (Nlrp3)*, interleukin-* (IL-) 1beta,* and* IL-18* expression were measured by qRT-PCR ((f), (g), and (h), resp.). Detection of caspase-1 activation was achieved by a colorimetric assay, as described in Materials and Methods (i). Results are mean ± s.e.m. from six (a, c), five (f, g, h), four (b), or three (e, i) independent experiments; (c) and (i) were performed in duplicate. Comparisons between lipopolysaccharide- (LPS-) treated N9 cells and nontreated cells (control) were made via two-tailed Student's *t*-test for all the parameters, except for (c, e), where unpaired *t*-test with Welch's correction was applied. ^*∗*^ *p* < 0.05, ^*∗∗*^ *p* < 0.01, and ^*∗∗∗*^ *p* < 0.001 versus nontreated cells (control). Scale bar represents 20 *µ*m.

By investigating the signaling pathway mediated by TLR/NF-*κ*B activation, we found that TLR4 was translocated to the membrane of microglial cells incubated with LPS, as demonstrated by an increased expression of the glycosylated form (*p* < 0.01, Figure 5(a)). We also noticed an increased expression of TLR2 (*p* < 0.05, Figure 5(b)). Consequently, NF-*κ*B was translocated into the nucleus, as demonstrated by the ~2-fold increase in protein expression in the nuclear fraction (*p* < 0.05, Figure 5(c)) and by the elevated staining of LPS-treated N9 microglia nuclei when compared to control samples (Figures 5(d) and 5(e)). NF-*κ*B transactivation was also demonstrated in reactive microglia primary cultures [39] and to be implicated in inflammasome activation [47, 48] that is associated with the maturation of the microglial proinflammatory cytokines IL-1*β* and IL-18 by caspase-1 [16, 49]. Hence, the next step was to evaluate the cascade of such events in the N9 microglia exposed to LPS. As depicted in Figure 5, LPS significantly enhanced the expression of* Nlrp3* inflammasome (Figure 5(f), *p* < 0.001) as well as of* IL-1beta* (Figure 5(g), *p* < 0.05) and* IL-18* (Figure 5(h), *p* < 0.05) in N9 cells. The activation of inflammasome was also observed by treating N9 cells [50], immortalized microglia [51], and cultured primary microglia (our unpublished data) with amyloid-beta peptide. Moreover, the high caspase-1 activity we observed (Figure 5(i), *p* < 0.01) further indicates an increased capacity to mediate the cleavage of IL-1*β* and IL-18 proforms [49].

**Figure 6:**
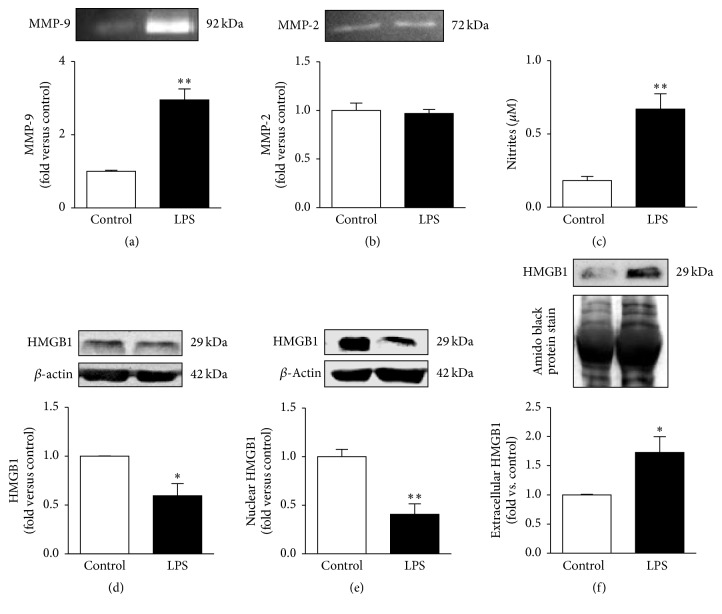
M1 polarized N9 microglia have low intracellular content of HMGB1 and increased secretion of MMP-9, NO, and HMGB1 to the cell milieu. Extracellular medium was assessed for matrix metalloproteinase-9 (MMP-9) (a) and MMP-2 (b) activities by the gelatin zymography assay and results were expressed as fold versus nontreated cells (control). Nitrites that reflect nitric oxide (NO) production were assessed by the Griess reaction followed by Microplate Reader for Absorbance Assays (c). High mobility group box 1 (HMGB1) expression was determined in total extracts (d), nuclear fraction (e), and extracellular media (f) by Western blot analysis. Results are mean ± s.e.m. from six (a, b, d, e) or four (c, f) independent experiments. Nitrites measurement (c) was performed in duplicate. Comparisons between lipopolysaccharide- (LPS-) treated N9 cells and nontreated cells (control) were made via two-tailed Student's *t*-test (e) or unpaired *t*-test with Welch's correction (a, c, d, f). ^*∗*^ *p* < 0.05 and ^*∗∗*^ *p* < 0.01 versus nontreated cells (control).

M1 polarized microglia is also related to the generation of other inflammatory mediators, such as ROS and MMPs [12, 13]. Actually, we found elevated levels of MMP-9 (~3-fold, *p* < 0.01), but not of MMP-2 (Figures 6(a) and 6(b), resp.), which may derive from the observed NF-*κ*B activation (Figures 5(c)–5(e)) demonstrated to be a regulator of MMP-9 gene expression, but not of MMP-2 [52]. NO is considered a signaling molecule and a proinflammatory mediator, as well. Our studies showed that the concentration of nitrites, indirectly indicating the level of NO, was likewise markedly enhanced in the extracellular medium (Figure 6(c)).

Proinflammatory effects of LPS were shown to be enhanced by the endogenous danger signal molecule HMGB1 released by necrotic cells [53]. The majority of the studies have reported HMGB1 effects on macrophages and microglia activation [18, 54], but only a few report its increased expression in activated microglia, both in the postischemic brain [55] and in the reactive primary microglia cultures [39]. In addition, we recently found more than a 2-fold increase in both gene and protein expression of HMGB1 in N9 cells treated with 1 *µ*M amyloid-beta peptide [50]. Therefore, we examined the pattern of cellular and extracellular distribution of HMGB1 in our inflammatory N9 cells. Surprisingly, we observed a reduced HMGB1 protein expression in cell lysates of microglia activated by LPS and in nuclear extracts (Figures 6(d) and 6(e)). However, once HMGB1 was shown to be massively released into the extracellular environment in pathological conditions [54], we decided to evaluate its presence in cellular supernatants. In accordance with this, HMGB1 was found to accumulate in culture medium after incubating N9 microglial cells with LPS for 24 h (~2-fold increase, *p* < 0.05), as depicted in Figure 6(f). These results indicate that HMGB1 is rapidly released into the extracellular space after LPS-induced microglial activation.

### 3.4. Differential Expression of Inflamma-miRs in M1 Polarized N9 Microglia by LPS Is Recapitulated in Cell-Derived Exosomes

To identify miRNA subtypes subsequent to N9 cell polarization by LPS we evaluated the expression of specific inflammatory miRNAs associated with microglia activation, namely, mmu-miR-155-5p (miR-155), related to M1 phenotype, hsa-miR-146a-5p (miR-146a) linked to repair, and resolution and hsa-miR-124-3p (miR-124), expressed in surveillant and anti-inflammatory microglia. Such inflamma-miRs were previously identified in reactive cultured microglia [39] and both miR-155 and miR-146a found to be elevated in N9 cells upon treatment with amyloid-beta peptide [50].

**Figure 7:**
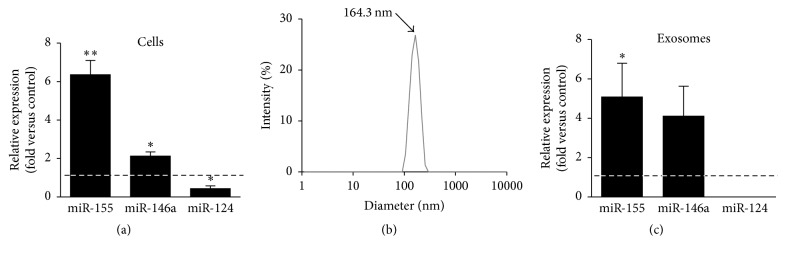
M1 polarized N9 microglia display upregulated levels of miR-155 and miR-146a, as well as downregulated miR-124, which are recapitulated in cell-derived exosomes. N9 cells were collected after incubation with lipopolysaccharide (LPS) and miR-155, miR-146a, and miR-124 expression were evaluated by qRT-PCR, both in cells (a) and in exosomes (c). Results are mean ± s.e.m. from five independent experiments. Comparisons between LPS-treated N9 cells and nontreated cells (control) were made via two-tailed Student's *t*-test. ^*∗*^ *p* < 0.05 and ^*∗∗*^ *p* < 0.01 versus nontreated cells (control). Vesicles isolated from the extracellular media of N9 cells were characterized in terms of their size by dynamic light scattering (b).

As displayed in Figure 7, microglia stimulation with LPS induced an elevated expression of miR-155 (~6-fold, *p* < 0.01) and of miR-146a (2-fold, *p* < 0.05), while promoting the downregulation of miR-124 expression (0.4-fold, *p* < 0.05). These data support the acquisition of a M1 phenotype in microglia activated by LPS with the loss of their neuroprotective properties and sustained upregulation of proinflammatory features by the miR-155 regulation networks. Intriguingly, the overexpression of miR-146a may constitute a mechanism of regulating the inflammatory response [56], exemplifying the complexity of the M1/M2/M0 landscape. In addition, we found that N9 cells were able to release exosomes of approximately 160 nm diameter, as determined by DLS analysis (Figure 7(b)). We observed that these extracellular vesicles carried the same miRNAs of the M1 polarized cell, demonstrating that such exosomes recapitulate the miRNA cargo of the cell of origin. Moreover, since miR-124 was only detectable in nonstimulated N9 cells (control), we may hypothesize that miR-155 and miR-146a are the most implicated in promoting the phenotypic conversion of adjacent cells.

## 4. Discussion

In this study, we have profiled N9 microglia activation upon LPS stimulation at cellular, functional, and molecular levels, by considering either gene or protein expression of inflammatory/anti-inflammatory associated markers. In addition, we not only characterized the different faces of microglial response associated with the M1 polarization but also explored the involvement of new inflammatory players. Data support LPS-induced microglia M1 phenotype with the activation of the inflammatory cascade mediated by TLR/NF-*κ*B signaling pathway in N9 cells. More interestingly, we provide new evidence indicating decreased migration ability but increased phagocytosis with the involvement of MFG-E8 production and release. In addition, our study is innovative in evidencing that LPS triggers the upregulation of the NLRP3 inflammasome complex in N9 cells and leads HMGB1 release to the extracellular medium together with that of MMP-9. For the first time we obtained upregulation of miR-155 and miR-146, along with a reduced expression of miR-124 in the N9 cells treated with LPS, as previously observed in macrophages/microglia M1 polarized cells [24], and we showed that a similar representative profile occurs in exosomes isolated from LPS-treated N9 cells supernatants.

N9 cell line has been widely used in the evaluation of microglial functions in different conditions, as an alternative to primary cell cultures, due to increased yield and homogeneity of cells in culture. More importantly, no differences were found between N9 cells and microglia primary cultures relative to the activation of inflammatory mediators by LPS [21]. Previous separated studies evidenced an assembly of effects induced by LPS that included NF-*κ*B activation [57], amoeboid morphology with process retraction [58, 59], release of proinflammatory mediators [4, 58], low migratory capacity [33], and increased phagocytic ability [60]. Nevertheless, there is still limited information concerning the activation pattern of these cells and the combined events implicated in the phenotypic transition towards M1 polarization, which are mandatory when intrinsic characteristics of microglia are to be explored and therapeutic agents to be tested. Moreover, no inflammasome activated complex was ever described, nor were inflamma-miR profiling and exosome cargo identified. In addition, only our data report the increased expression of HMGB1 in amyloid-beta peptide-treated N9 cells [50], as well as its nucleocytoplasmic shuttling and further release in LPS-treated cells, as we describe in this study. We first settled that our N9 microglia predominantly switched to the M1 subtype in the presence of LPS, as described for macrophages and microglia primary cultures [12, 13]. Using established markers that allow the differentiation between M1 and M2 activated cells (for review see [8]), we observed that the M1-markers* Nos2* and* Mhc-II* were upregulated while the M2-markers* Arg1* and* Fizz1* were downregulated after LPS exposure. In conformity, we also observed downregulation of CX3CR1, a condition identified in M1 monocyte-macrophages [61] and in primary microglia exposed to LPS [62]. Moreover, low CX3CR1 is associated with marked neuronal loss after systemic inflammation [11] and facilitates sepsis-induced immunosuppression resulting from monocyte inability to recognize CX3CL1 and kill pathogenic microorganisms [63]. Therefore, the decrease of CX3CR1 in our model may relate to the persistent microglia activation.

The increased number of amoeboid N9 microglia and the reduction of ramified cells are features of microglia activation and were observed after treatment with LPS [58, 59]. Increased proliferation rate of microglia by LPS, as we obtained in N9 cells based on the nuclear appearance of the Ki-67 marker and upregulation of* Cd11b* expression, was previously noticed in BV2-stimulated microglia [64]. Nevertheless, in vivo data do not sustain the presence of proliferating microglia during systemic inflammation [65], though it was evidenced during ischemia [66].

The ability of microglia to sense distant signals and be attracted to them, a function designated by chemotaxis, is orchestrated by multiple chemotactic compounds released at the site of damage, either by injured cells or by pathogenic organisms. As claimed by others [3], ATP showed to be a chemotactic agent and promoted the migration of N9 microglia, a property that was however reduced after LPS treatment, probably as a consequence of the diminished processes that compromise the migratory capacity towards a chemoattractant signal [9]. Indeed, cell migration to lesion sites is faster than morphological changes associated with microglia activation, in which purines such as ATP can no longer attract and may even repel them [67]. Such alterations may relate to changing of surface markers such as P2Y_12_ upon LPS exposure [68]. In fact, P2Y receptors were previously associated with microglia chemotactic function to ATP [69]. Reduced ability to migrate towards LPS was also a feature of our M1 polarized N9 cells, although there is some controversy on the ability of LPS to induce or inhibit migration [45, 70, 71], since it may depend on LPS concentration and on mediators released by the activated microglia near the site of injury [72].

Phagocytosis is another microglial function that is extremely important to their neuroprotective properties, required not only for synaptic plasticity but also for clearance of cellular debris and elimination of pathogenic organisms [46]. Our results indicate that LPS increases N9 microglia phagocytosis, as previously described for LPS-treated BV2 cells, as well as primary cultures of microglia and macrophages [73]. In vivo studies showed that LPS administration increases microglia phagocytosis of viable neurons during development, inflammation, and neuropathology, a property denominated as phagoptosis [74]. A new finding was the increased expression of the phagocytosis-associated protein MFG-E8, together with its release, when the N9 cells were treated with LPS. There are still controversial data concerning the protective/noxious role of MFG-E8. On one hand, MFG-E8 has been associated with microglia anti-inflammatory properties, as demonstrated by Spittau et al. [75], when the cells were stimulated by TGF-*β*, in the MGF-E8^−/−^ mice showing an increased proinflammatory response [19], as well as in the septic mouse presenting downregulation of MFG-E8 levels [76]. On the other hand, Liu and colleagues [77] have demonstrated that MFG-E8 overexpression occurred concomitantly with TNF-*α* and IL-1*β* upregulation. Thus, given the increased expression of intracellular and released MFG-E8 found in our model, we hypothesize that MFG-E8 upregulation is typical of M1 polarized microglia.

Considering the inflammatory signaling cascades, the activation of TLR4/2-NF-*κ*B pathway is consistent with the proinflammatory response triggered by LPS [57, 58]. The role of inflammasome is poorly understood in microglia activation pattern. Our data show for the first time that expression of NLRP3, IL-1*β*, and IL-18 mRNA increases by LPS exposure and may relate to the activation of caspase-1. Bauernfeind and colleagues [47] proposed that NF-*κ*B induces mRNA expression of NLRP3, which is further required for the formation of inflammasome complexes. Others have reported the activation of NLRP3 complexes and recruitment of caspase-1, together with IL-18/IL-1*β* release, in primary microglia [78] and in macrophages [49] stimulated by infection-associated agents. Our results are also consistent with works in the BV-2 cell line and in rat primary microglia reporting that microglia exposed to LPS show increased generation of redox molecules (NO) and iNOS activation [57, 58], as well as enhanced MMP-9 expression [70] and activity [79]. Most interesting, TLR2-dependent NO expression was shown to be necessary for microglial MMP-9 expression [80]. Although it has been shown that MMP-2 may be also involved in glial cell activation [81] and our recent studies indicate increased release of MMP-2 into the extracellular media in microglia treated with amyloid-beta peptide (unpublished results), we did not observe any variation between LPS-stimulated and nonstimulated cells (control) in terms of MMP-2 release. This difference between the results found for MMP-9 and MMP-2 may be explained by the existence of specific binding sites for NF-*κ*B in MMP-9, which are lacking in MMP-2 promoter region, as reported by Fanjul-Fernández et al. [52].

We next explored the expression of the alarmin HMGB1 in the context of inflammation in our culture model, since it has been described to be a signal released to the extracellular environment that leads to inflammatory mechanisms through the activation of RAGE and TLR2/TLR4 receptors [18]. We are the first to report that the decrease in its nuclear and cytoplasmic content in N9 microglia after the LPS stimulus is most likely due to its substantial release to the cell supernatants. Actually, HMGB1 is released from the cells after translocation from the nucleus to the cytoplasm [82], indicating that secretion of HMGB1 may be associated with inflammasome complex activation [17].

The influence of miRNA in microglia-mediated immune response was recently reviewed [83] and abnormal expression of inflamma-miRs showed them to contribute to chronic proinflammatory status and to be a significant mortality risk factor [84]. MiRNAs are small noncoding RNA molecules that are 18–25 nucleotides in length involved in the regulation of gene expression. Main inflammatory miRNAs are miR-155, miR-21, miR146a/b, and miR-124 [85]. We found upregulation of miR-155 and miR-146, together with a reduced expression of miR-124, in N9 microglia upon LPS exposure. This pattern corroborates the increased representation of polarized M1 microglial N9 by LPS over the M2 polarization, as described by several authors [24, 36]. Freilich and colleagues [22] found a similar expression profile in primary cultured microglia upon a short-time incubation with LPS (4 h). Targets of miR-155 are the suppressor of cytokine signaling 1 (SOCS1), CCAAT/enhancer-binding protein alpha (C/EBP*α*), and Smad2 [24, 36] which are important players in anti-inflammatory response. As such, increased expression of miR-155 potentiates inflammation and sustains microglia activation and resulting neurotoxicity. We have also demonstrated here that incubation with LPS promotes upregulation of miR-146a in N9 microglia. It is possible that such effect results from the TLR2/TLR4 activation, as described for BV-2 and EOC 13.31 cell lines [86]. Others indicate that miR-146a regulates NF-*κ*B signaling pathway by directly targeting IRAK1 and tumor-necrosis-factor-receptor-associated factor 6 (TRAF6), proteins involved in the transduction pathway of NF-*κ*B transactivation by immune stimulation [87]. However, in our model, the upregulation of miR-146a at 24 h incubation with LPS was not able to suppress inflammatory response. This inability was similarly observed when 72 h treatment was assayed (data not shown). Interestingly increased expression of miR-146a may be related to LPS tolerance, previously observed in monocytes and suggested to act as a tuning mechanism to prevent an overstimulated inflammatory state [88]. In what concerns miR-124, known to promote microglia quiescence [89], it was revealed to switch cell polarization from M1 to the M2 phenotype [90] in various subsets of monocyte cells and microglia [26]. This finding indicates that the decreased expression of miR-124 that we found in our model is consistent with the phenotype M1 preponderance. Our data also indicate that the expression profile of inflamma-miRs in exosomes recapitulates the one found in the cells, which suggests that M1 polarized microglia is able to directly transport miRNAs to an adjacent cell and modulate its phenotype, besides the release of soluble inflammatory signals. As a consequence, the proinflammatory environment observed in many neurotoxic situations may be sustained through the transfer of the microglia phenotype associated miRNAs from cells into exosomes.

## 5. Conclusions

**Figure 8:**
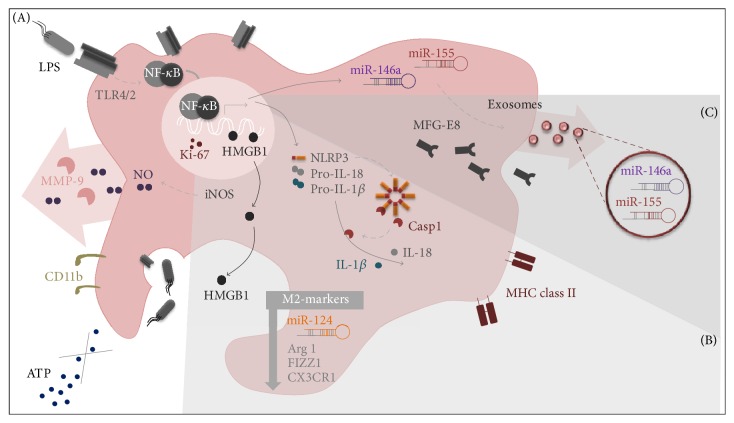
Schematic representation of inflammatory players implicated in lipopolysaccharide- (LPS-) induced M1 polarization of N9 microglial cells and of exosome upregulated microRNAs. (A) We first studied functions and pathways commonly described for microglia classical activation. We observed that LPS causes a moderate degree of apoptosis in the N9 cells and a switch from the ramified to an amoeboid cell shape. LPS-stimulated cells lose the ability to migrate towards ATP but show increased phagocytic ability, as revealed by the elevated number of latex beads ingested. LPS also triggers microglia proliferation as indicated by the increased number of* Cd11b* positive cells and Ki-67 stained nuclei. Inflammatory events are mediated through the signaling pathway involving the toll-like receptor 4 (TLR4)/TLR2 and nuclear factor kappa B (NF-*κ*B) in LPS-treated N9 microglia and implicate the upregulation of microRNA-155 (miR-155) and miR-146a as well as the release of nitric oxide (NO) and matrix metalloproteinase-9 (MMP-9) to the extracellular milieu. (B) Our study provided new evidence that N9 cells treated with LPS have increased expression of inflammasome complex comprehending the upregulation of interleukin-* (IL-) 1beta*,* IL-18,* and NOD-like receptor family pyrin domain containing 3* (Nlrp3)*, together with enhanced caspase-1 activation. Increased expression of* Nos2* and major histocompatibility complex class II* (Mhc-II)* (markers of M1 polarization or classically activated microglia), in conjunction with decreased expression of arginase 1* (Arg1)*, found in inflammatory zone 1* (Fizz1),* miR-124 (markers of M2 polarization or alternatively activated microglia), and reduced CX3C chemokine receptor 1 (CX3CR1) expression corroborate the acquisition of a prevalent M1 phenotype in microglial cells exposed to LPS. (C) Finally, we described for the first time that increased MFG-E8 expression and release are induced in M1 polarized microglia and that miRs expression profile is recapitulated in cell-derived exosomes, further supporting M1 polarization in LPS-treated N9 cells and dissemination of inflammatory mediators by extracellular vesicles.

Overall, the present data integrate an ensemble of signaling cascades related to the M1 path activation of N9 microglial cells, together with innovative data pointing to inflammasome complex activation, downregulation of the neuronal fractalkine receptor CX3CR1, and reduction of miR-124 upon incubation with LPS. New findings also include the LPS-induced increase in the expression of MFG-E8, miR-155, and miR-146a, together with raised secretion of HMGB1 and MMP-9, as schematically represented in Figure 8. Our study firstly identified that the delivery of miR-155 from microglia to adjacent cells may be mediated by exosomes, as previously observed for dendritic cells in response to endotoxin [32]. Our model of LPS-induced M1 polarization of N9 cells, by exploring new molecules and pathways implicated in such activation, enhanced our understanding on the neurodegenerative processes associated with microglia inflammation, while identifying promising biomarkers to be used in neurological disorders, in particular exosomes and their cargo in miR-155. Finally, our study further suggests that targeting NLRP3 inflammasome, HMGB1 signaling, and miR-155 transfer to exosomes may be suitable therapeutic approach to restrain neuroinflammation in disorders whereby microglia activation has a critical role.

## Supplementary Material

Supplementary Figure S1 describes the morphological analysis that was performed by using immunostaining with anti-Iba1 in N9 microglial cells, and comparison with primary cultures of microglia from mice brain. Table S1 lists the primers used in qRT-PCR. Table S2 lists the primary antibodies used in Western Blot analysis.

## Figures and Tables

**Table 1:** N9 microglia exposed to LPS show increased cell death as indicated by Guava Nexin assay.

	Viable cells (%)	Early apoptosis (%)	Late apoptosis/necrosis (%)
Control	84 ± 8.3	10.6 ± 4.2	5.0 ± 3.0
LPS	66.9 ± 4.5^*∗*^	23.2 ± 7.6^*∗*^	10.3 ± 5.0^*∗*^

Phycoerythrin-conjugated annexin V (V-PE) and 7-aminoactinomycin D (7-AAD) (Guava Nexin Reagent, #4500-0450, Millipore) were used to determine the percentage of viable, early-apoptotic, and late-apoptotic/necrotic cells by flow cytometry. After incubation, cells were trypsinized, added to extracellular media, and then stained with annexin V-PE and 7-AAD. Samples were analyzed on a Guava easyCyte 5HT flow cytometer (Guava Nexin Software module, Millipore). Three distinct populations of cells were identified: viable cells (annexin V-PE and 7-AAD negative), early-apoptotic cells (annexin V-PE positive and 7-AAD negative), and late stages of apoptosis/necrosis (annexin V-PE and 7-AAD positive). ^*∗*^ *p* < 0.05 versus nontreated cells (control).
