# Supplementary material for: Exploring New Inflammatory Biomarkers and Pathways during LPS-Induced M1 Polarization
Source: Mediators Inflamm. 2016 Dec 21;2016:6986175. doi: 10.1155/2016/6986175 (PMC5209629; doi:10.1155/2016/6986175)
Supplement: Supplementary file 1 — Supplementary Figure S1 describes the morphological analysis that was performed by using immunostaining with anti-Iba1 in N9 microglial cells, and comparison with primary cultures of microglia from mice brain. Table S1 lists the primers used in qRT-PCR. Table S2 lists the primary antibodies used in Western Blot analysis. [file 6986175.f1.pdf]

Fig. S1

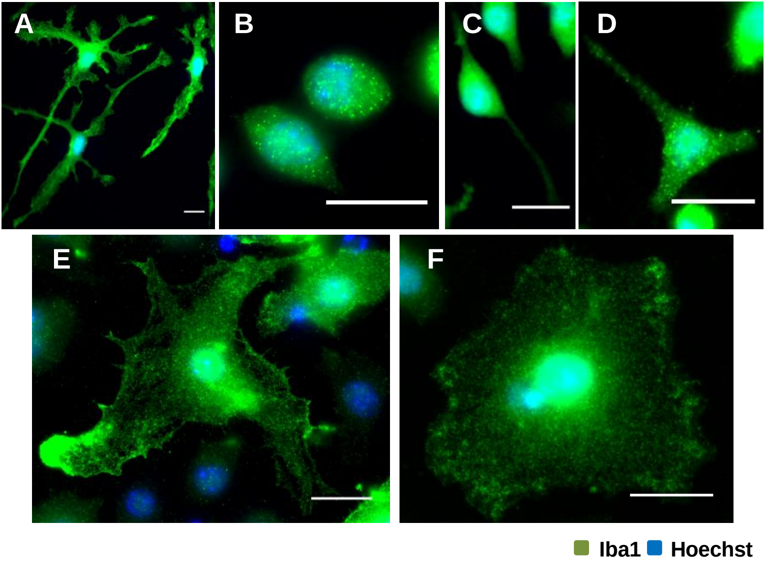

Fig. S1. N9 microglial cells show lower number and length of ramifications than primary cultured microglia from mice cortical brain and a combination of distinct morphologies. Morphological analysis was performed by immunocytochemistry using anti-Iba1, as indicated in methods. Nuclei were stained with Hoechst (blue fluorescence). Primary cultures of microglia isolated from mice cortical brain were stained with Iba-1 and used for comparative purpose (A). Different morphologies observed in N9 cells included round/oval (B), ramified with two (C) or three (D) ramifications, amoeboid with thicker branches (D) and amoeboid with absent ramifications (E). Representative results of one experiment are shown and scale bar represents 20 µm.

Table S1 – List of primers used in qRT-PCR

Gene	Forward Primer Sequence	Reverse Primer Sequence
beta-actin	5'-GTCCCGGCATGTGCAA-3'	5'-AGGATCTTCATGAGGTAGT-3'
Nos2	5'-ACCCACATCTGGCAGAATGAG-3'	5'-AGCCATGACCTTTCGCATTAG-3'
Mhc-II	5'-TGGGCACCATCTTCATCATTC-3'	5'- GGTCACCCAGCACACCACTT-3'
Arg1	5'-CTTGGCTTGCTTCGGAACTC-3'	5'-GGAGAAGGCGTTTGCTTAGTTC-3'
Fizz1	5'-GCCAGGTCCTGGAACCTTTC-3'	5'-GGAGCAGGGAGATGCAGATGAG-3'
Cx3cr1	5'-TCGTCTTCACGTTCGGTCTG-3'	5'-CTCAAGGCCAGGTTCAGGAG-3'
Cd11b	5'-CAGATCAACAATGTGACCGTATGGG-3'	5'-CATCATGTCCTTGTACTGCCGCTTG-3'
Nlrp3	5'-TGCTCTTCACTGCTATC AAGCCCT-3'	5'-ACAAGCCTTTGCTCCAGACCCTAT3'
Il-1beta	5'-CAGGCTCCGAGATGAACAAC-3'	5'-GGTGGAGAGCTTTCAGCTCATA-3'
Il-18	5'-TGGTTCCATGCTTTCTGGACTCCT-3'	5'-TTCCTGGGCCAAGAGGAAGTG-3'

miRNA	Primer Sequence
**mmu-miR-155-5p**	5'-UUAAUGCUAAUUGUGAUAGGGGU-3'
**hsa-miR-146a-5p**	5'-UGAGAACUGAAUUCCAUGGGUU-3'
**hsa-miR-124-3p**	5'-UAAGGCACGCGGUGAAUGCC-3'

Table S2 – List of primary antibodies used in Western Blot

Primary antibody	Species	Reference	Dilution
**β-actin**	mouse	A5441, Sigma-Aldrich, St. Louis, MO, USA	1:5000
**CX3CR1**	rabbit	sc-30030, Santa Cruz Biotechnology®, CA, USA	1:100
**MFG-E8**	rabbit	sc-33546, Santa Cruz Biotechnology®, CA, USA	1:175
**TLR4**	rabbit	sc-10741, Santa Cruz Biotechnology®, CA, USA	1:100
**TLR2**	rabbit	sc-10739, Santa Cruz Biotechnology®, CA, USA	1:100
**NF-κB**	rabbit	sc-372, Santa Cruz Biotechnology®, CA, USA	1:500 (or 1:200 for nuclear extracts)
**HMGB1**	mouse	651402, BioLegend,San Diego, CA, USA	1:200
